# Bacoside A: Role in Cigarette Smoking Induced Changes in Brain

**DOI:** 10.1155/2015/286137

**Published:** 2015-08-27

**Authors:** G. Vani, K. Anbarasi, C. S. Shyamaladevi

**Affiliations:** Department of Biochemistry, University of Madras, Guindy Campus, Chennai 600 025, India

## Abstract

Cigarette smoking (CS) is a major health hazard that exerts diverse physiologic and biochemical effects mediated by the components present and generated during smoking. Recent experimental studies have shown predisposition to several biological consequences from both active and passive cigarette smoke exposure. In particular, passive smoking is linked to a number of adverse health effects which are equally harmful as active smoking. A pragmatic approach should be considered for designing a pharmacological intervention to combat the adverse effects of passive smoking. This review describes the results from a controlled experimental condition, testing the effect of bacoside A (BA) on the causal role of passive/secondhand smoke exposure that caused pathological and neurological changes in rat brain. Chronic exposure to cigarette smoke induced significant changes in rat brain histologically and at the neurotransmitter level, lipid peroxidation states, mitochondrial functions, membrane alterations, and apoptotic damage in rat brain. Bacoside A is a neuroactive agent isolated from *Bacopa monnieri*. As a neuroactive agent, BA was effective in combating these changes. Future research should examine the effects of BA at molecular level and assess its functional effects on neurobiological and behavioral processes associated with passive smoke.

## 1. Introduction 

Cigarette smoking is an intractable and preventable public health problem. It is an important risk factor involved in the pathogenic pathways of a variety of disorders. The WHO has declared global tobacco epidemic and planned “Framework Convention for Tobacco Control” [1]. Tobacco smoke is a toxic air contaminant and secondhand tobacco smoke (SHS) is a formidable health hazard [2]. Epidemiological studies show increased risk for behavioral and cognitive problems and a greater incidence of mental disorders in children exposed to environmental tobacco smoke [3–5]. Prenatal maternal exposure to cigarette smoke has been well documented to induce neurological as well as many other lasting health effects [6, 7]. Etiological evidences support the involvement of neurotransmitter systems, oxidative and nitrogen stress, mitochondrial dysfunction, and neurogenetic and epigenetic changes in secondhand/passive smoking induced brain changes and the associated pathways have been extensively reviewed [8–11].

Despite the significant health impacts arising from passive smoking, little attention is paid to combat the neurological changes associated with it. This review critically examines and summarizes the study made on the neuroprotective role of BA in rats exposed to passive cigarette smoke and its sequelae with focus on the neurotransmitter systems, oxidative and lipid peroxidative, mitochondrial dysfunction, and apoptotic changes in rat brain. These results can be integrated with other theories in holistically combating passive smoking induced neurological changes.

## 2. Physical and Biochemical Properties of Cigarette Smoke

Cigarette smoke is divided into two phases: a tar phase and a gas phase. The tar or particulate phase is defined as the material that is trapped when the smoke stream is passed through the Cambridge glass-fiber filter that retains 99.9% of all particulate material with a size ~0.1 m [12]. The gas phase is the material that passes through the filter. The particulate (tar) phase of cigarette smoke contains ~10^17^ free radicals/g, and the gas phase contains ~10^15^ free radicals/puff [12]. The radicals associated with the tar phase are long-lived (hours to months), whereas the radicals associated with the gas phase have a shorter life span (seconds) [12–14].

Cigarette smoke that is drawn through the tobacco into an active smoker's mouth is known as mainstream smoke (MS) and the smoke emitted from the burning ends of a cigarette is the sidestream smoke (SS). Mainstream cigarette smoke comprises 8% of tar and 92% of gaseous components [12]. Environmental tobacco smoke (ETS) results from the combination of sidestream smoke (85%) and a small fraction of exhaled mainstream smoke (15%) from smokers [13]. Importantly, the concentration of numerous toxins is dramatically (up to 100-fold) elevated in SS when compared with MS, and the complex mixture of toxins is attributed rather to a specific component of cigarette smoke to the potential adverse impact of passive smoke on health [15].

Aside from specific chemical constituents, certain physicochemical properties of smoke may participate in disease processes. The pH of the smoke affects the site and degree of nicotine absorption as well as the smoker's depth of inhalation. The oxidation-reduction state of the smoke is important because oxidants influence the maturation of cholesterol-laden plaques in the coronary arteries and other blood vessels. In short, cigarette smoke is far more than a triad of tar, nicotine, and carbon monoxide [16].

Although SS and MS smoke have qualitatively similar chemical compositions, the respective quantities of individual smoke constituents are different [17]. The exposure to SS smoke depends on the distance from the burning cigarette and conditions of ventilation; the higher concentrations of certain toxic and carcinogenic chemicals in SS smoke result in measurable levels of these chemicals in nonsmokers exposed to ETS [18].

## 3. Pharmacodynamics of Smoking

Although most of the toxicity of smoking is related to other components of cigarette smoke, it is primarily the pharmacologic effects of nicotine that produce the addiction to tobacco. An understanding of how nicotine produces addiction and influences smoking behavior provides a necessary basis for optimal smoking cessation intervention. Cigarette smoke contains 10–14 mg of nicotine [19], of which 1–1.5 mg is absorbed systemically in the lungs through inhalation [20]. Nicotine rapidly enters the pulmonary venous circulation, reaches the brain within 10–20 s, and readily diffuses into brain tissue [21] and binds to nicotine acetylcholine receptors (nAChRs) [22]. Acutely, cigarette smoking induces positive reinforcing effects, including mild euphoria, heightened arousal, reduced appetite, and reduced stress, anxiety, and pain [23].

Nicotine in cigarette smoke is alkaline and readily crosses the blood brain barrier. It mediates the stimulation of mesolimbic dopamine system. It is also involved in modulating other classical neurotransmitters in the brain including catecholamines, serotonin, GABA, and glutamate [24]. It induces addiction as it shares many properties of psychostimulant drugs such as cocaine and amphetamine [25]. With repeated exposure to nicotine, toxicity gives way to tolerance and addiction. Overtime tolerance is accompanied by increased intake of nicotine to attain the same effects initially taken which leads to physical dependence, compelling higher intake, lest it induces withdrawal symptom. This leads to reinforcement mechanisms, finally leading to addiction [26]. Substances other than nicotine present in cigarette smoke are also involved in mediating the harmful effects in nervous system. Other tobacco smoke constituents affect the structural and functional integrity of blood brain barrier [27, 28].

## 4. Neuropharmacological Effects of Cigarette Smoking 

Cigarette smoking is an important environmental aging accelerator [29] partly because it induces oxidative stress in multiple organs including the brain and is presented in many diseases, including cognition-related or neurodegeneration-related pathological changes [30]. This should be presented to demonstrate a direct linkage between smoking and cognitive impairment.

The incidence of cerebrovascular diseases (CVDs) increases with cigarette smoking, as cessation of smoking decreases its incidence [31]. Smoking is a modifiable risk factor for stroke [32] primarily due to hypertension [33]. Other neurological diseases for which smoking is a risk factor include hypoxia, cerebral ischemia, cerebral hemorrhage, brain infarction, subarachnoid hemorrhage, and tardive dyskinesia [34]. Neuroleptic Parkinsonism, resembling Idiopathic Parkinson's disease, is associated with smoking, as higher doses of nicotine exert an inhibitory effect [35]. Cerebral symptoms like brain atrophy and ataxia are exaggerated with smoking [36]. Reports also suggest that cigarette smoking is protective in the development of Parkinson's disease [37] but is an important risk factor in Alzheimer's disease [38], as it accelerates cognitive decline and dementia [39].

## 5. Role of Passive Smoking

The evidence that active smoking is a risk factor for cardiovascular disease (CAD) and the leading cause of preventable death is overwhelming. However, exposure to passive cigarette smoke also exerts detrimental effects on vascular homoeostasis [40]. Importantly, most of these effects appear to be characterized by a rapid onset. For example, the relatively low doses of toxins inhaled by passive smoking are sufficient to elicit acute endothelial dysfunction, and these effects may be related, at least in part, to the inactivation of nitric oxide (NO). Moreover, passive smoking may directly impair the viability of endothelial cells and reduce the number and functional activity of circulating endothelial progenitor cells. In addition, platelets of nonsmokers appear to be susceptible to proaggregatory changes with every passive smoke exposure. Overall, passive smoke induces oxidative stress and promotes vascular inflammation. Apart from vasoconstriction and thrombus formation, however, the myocardial oxygen balance is further impaired by adrenergic stimulation and autonomic dysfunction [41]. These data strongly suggest that passive smoking is capable of precipitating acute manifestations as it increases the odds of developing cognitive impairment [42] and 3-fold increase in the risk for dementia, causing neurofibrillary changes depictive of Alzheimer's disease [43].

Hence, one generally overlooked factor contributing to the escalation of tobacco abuse is passive smoking. Nicotine from secondhand smoke exposure results in an increase in plasma nicotine concentration of ~0.2 ng/mL and amounts to substantial brain *α*4*β*2 nAChR occupancy (19%) in both smokers and nonsmokers compared with 0.87 ng/mL and 50% *α*4*β*2 nAChR occupancy from actively smoking one cigarette [44, 45]. Secondhand smoking is clearly linked to serious illnesses among nonsmokers including asthma, heart disease, sudden infant death syndrome, and cancer [46].

## 6. Pharmacological Intervention in Cigarette Smoking

With the available understanding of the biological effects of cigarette smoking many treatment strategies are available. The primary target is nicotine; hence nicotine replacement therapy or nicotine substitution in the form of chewing gums, transdermal patch, and spray are employed in smoking cessation aid [47–49]. Other pharmacological therapies include receptor antagonists and nicotinic antagonists (mecamylamine and chlorisondamine) [50]; opiate antagonists and naloxone and naltrexone [51]; nonreceptor antagonists [52]; serotonin uptake inhibitors (zimelidine and citalopram) and monoamine oxidase inhibitor [53]; and antidepressants [54, 55]. Angiotensin converting enzymes (ACE) inhibitors and calcium antagonists are also effective in combating smoking induced toxicity [56]. These drugs are mainly indicated to reduce the severity of tobacco withdrawal, but not as an aid to stop smoking [57].

Currently, the first line therapy for smoking cessation includes bupropion (amfebutamone), an atypical antidepressant that inhibits norepinephrine uptake and dopamine uptake [58]. The metabolite of bupropion, (2S,3S) hydroxybupropion, is an antagonist on the *α*4*β*2 (nAChR) [59]. Another agent is varenicline, which is a highly selective partial agonist of the *α*4*β*2 (nAChR) that stimulates dopamine release in the nucleus accumbens (nAC) but to a much less extent than nicotine itself [60].

These interventions are improbable and ineffective in rendering protection against secondhand smoke. Due to poor pharmacological management of passive smoking, it was hypothesized that intervening with a neuroactive agent can prevent or minimize neurological changes. Studies on supplementation with vitamins E, C, and A [61, 62]; antioxidants: glutathione, N-acetyl cysteine, and superoxide dismutase [63]; and fish oil, curcumin, and green tea [64–66] have reported to offer protection against smoking induced damages.

In this context, this study evaluated the effect of BA, an active constituent isolated from* Bacopa monnieri* against smoking induced damages in rat brain.* Bacopa monnieri* exerts neuropharmacological effects [67] and is effective in the treatment of mental illness and epilepsy [68]. Its biological effects include free radical scavenging [69]; vasodilatory [70, 71]; and mast cell stabilizing [72] activities. The various biological activities of BA have been reviewed in detail [73, 74].

Bacoside A is 3-(a-L-arabinopyranosyl)-O-b-D-glucopyranoside-10, 20-dihydroxy-16-keto-dammar-24-ene [75] and is the major chemical entity responsible for neuropharmacological effects and the nootropic action or antiamnestic effect of* Bacopa monniera*. Bacoside A cooccurs with bacoside B, the latter differing only in optical rotation and is probably an artefact produced during the process of isolating BA [76]. On acid hydrolysis, bacosides yield a mixture of aglycones, bacogenins A1, A2, and A3 [77], which are artefacts, two genuine sapogenins, jujubogenin and pseudojujubogenin, and bacogenin A4, identified as ebelin lactone pseudojujubogenin [78].

## 7. Methods

### 7.1. Isolation of Bacoside A

The plant* Bacopa monniera* was collected in and around Chennai, India, and authenticated by Dr. P. Brindha, Central Research Institute (Siddha), Chennai, India. The dammarane type triterpenoid saponin BA was isolated from the plant by the standard procedure. The purity of the isolated BA was identified by thin layer chromatography (TLC) and infrared (IR) spectrum analysis using standard BA [79].

### 7.2. Experimental Setup

Adult male albino rats of Wistar strain (120–200 g) were used for the present study. The rats were provided with standard pelleted rat feed and water ad libitum. They were acclimatized to the laboratory conditions and maintained under 12 h light and dark cycles. The experiments were carried out in accordance with the guidelines provided by the Institutional Animal Ethical Committee [79].

The animals were divided into four groups of 6 animals each. Group I: control. Group II (CS): rats exposed to cigarette smoke. Group III (BA): rats administered with BA (10 mg/kg bw/day, p.o.). Group IV (CS + BA): rats exposed to cigarette smoke and simultaneously administered with BA. Group II and Group IV rats were exposed to cigarette smoke, following a standard method as described [79] for a period of 12 weeks.

The rats were exposed to side stream cigarette smoke in whole body smoke exposure chamber. The rats were exposed twice daily as described [80, 81]. The experimental period lasted for 12 weeks. Drug control animals received aqueous suspension of BA in 1% gum acacia orally at a dosage of 10 mg/kg bw/day for 12 weeks, whereas experimental animals exposed to cigarette smoke (Scissors Standard Cigarette) were simultaneously administered with BA at the same dose. Control animals received a corresponding volume of the vehicle suspended in normal saline. The same brand of locally available cigarette was used throughout the experiment (Scissors Standard, W.D & H.O.Wills, Hyderabad Deccan Cigarette Factory). Control animals were subjected to the same handling and time in the smoke exposure chamber with air replacing smoke/air mixture. The composition of cigarette smoke was analyzed at Tamil Nadu Pollution Control Board, Chennai, and the constituents present are listed in Table 1.

## 8. Results and Discussion

### 8.1. Structural Brain Changes and Clinical Correlates

Cigarette smoking is associated with diverse structural changes in brain, probably as a consequence of toxicity or as an adaptive response, causing a reduction in integrity of cerebral white matter microstructure [82] and gray matter volumes [83, 84] and these changes appear correlated with the magnitude of cigarette exposure. Smoking induced structural changes in brain are associated with cognitive deficits [85] as well, with the integrity of white matter and glial proliferation [86]. In gross, the microstructural changes in key brain regions and white matter tracts have a negative impact in cigarette smokers.

**Figure 1:**
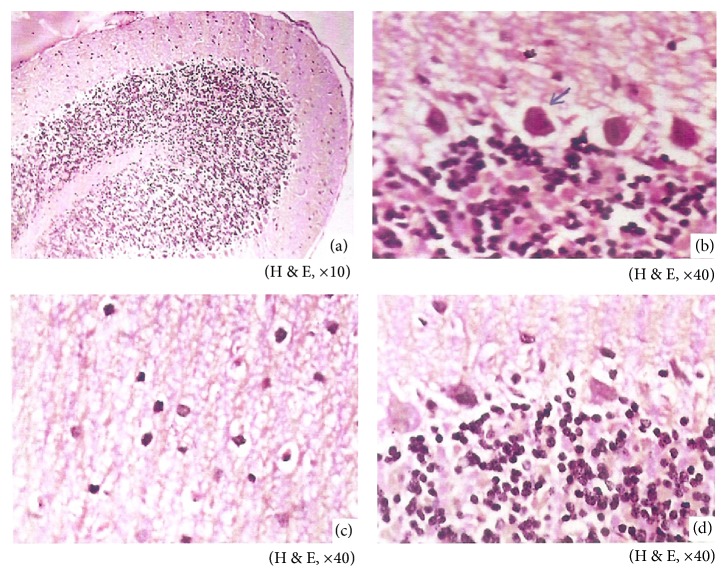
Sections of rat brain cerebellum. (a) Control rats showing normal architecture. (b) CS rats showing mild gliosis, edema, necrosis, and Purkinje cell damage. (c) BA rats showing normal architecture with no significant changes. (d) CS + BA rats showing normal morphology of Purkinje cells.

In the present study, histological changes were prevalent in brain of rats exposed to cigarette smoke that were inflammatory and edematous in the cerebrum (Figure 1). Smoking induced inflammatory changes were also marked by increased activity of CK-MB isoenzyme in serum [79], an early marker for pathological changes like cerebral damage [87]. 4-N-Methyl-N-nitrosamino-1-(3-pyridyl)-1-butanone (NNK), is a major nitrosamine present in substantial concentration in MS and SS that causes oxidative stress and triggers neuroinflammation in brain [88, 89]. Inflammation plays a pivotal role in extremely wide array of disease conditions ranging from viral diseases of CNS to neurodegenerative disorders. NKK mediated microglial activation leads to profound increase in inflammatory mediators. The inflamed milieu may cause neuronal damage [90]. A decrease in the inflammatory changes was noted in BA treated rats exposed to cigarette smoke, which could be due to the anti-inflammatory effect of BA [91] and the reduction in cerebral inflammatory changes in treated rats were also reflected in lowered levels of CK-MB as against untreated rats [79].

**Figure 2:**
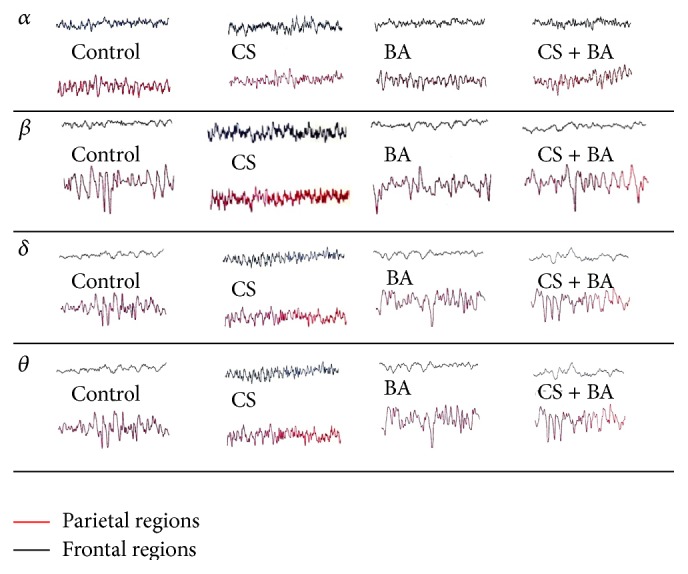
Electroencephalographic pattern of frontal and parietal regions of rat brain.

Electroencephalography (EEG) of rat brain monitored frontal and parietal regional changes in brain as electrical changes as *α*, *β*, *δ*, and *θ* waves. Cigarette smoke exposed rats presented depressed *δ* and increased *α* waves (Figure 2). A desynchronized and electrically active EEG pattern is noted in smokers [92]. Acute smoking accelerates dominant frequency fast waves *α* and *β* with a reduction in slow wave *δ* and *θ* waves illustrate a stimulant action [93], whereas chronic smoking induces less *α* wave and more *β* wave [94]. In rats treated with BA and exposed to cigarette smoke, the EEG pattern was devoid of desynchronization and lacked stimulatory wave, an effect also noted among cholinergic agonists: mecamylamine and scopolamine. This shows the anticholinergic effect of BA and effective against smoking induced stimulation of brain.

### 8.2. Neurotransmitter Systems

Neurotransmitters mediate diverse pharmacological effects on central and peripheral nervous system and participate in reinforcing, mood elevation, and cognitive functions [95]. A balance in their rate of synthesis and utilization constitutes the regulatory mechanism in neurotransmission. Smokers have positive effects like pleasure, arousal, and relaxation, as well as negative effects like depression and anxiety. The functional antagonism presented in cigarette smoking is related to desensitization of nAChR. Nicotine in cigarette smoke upregulates nAChR (pre- and postsynaptic), which in turn interacts with the noradrenergic, cannabinoid, dopaminergic, cholinergic, and serotonergic systems [96] and increases the levels of norepinephrine, dopamine, acetylcholine, and serotonin [97].

**Figure 3:**
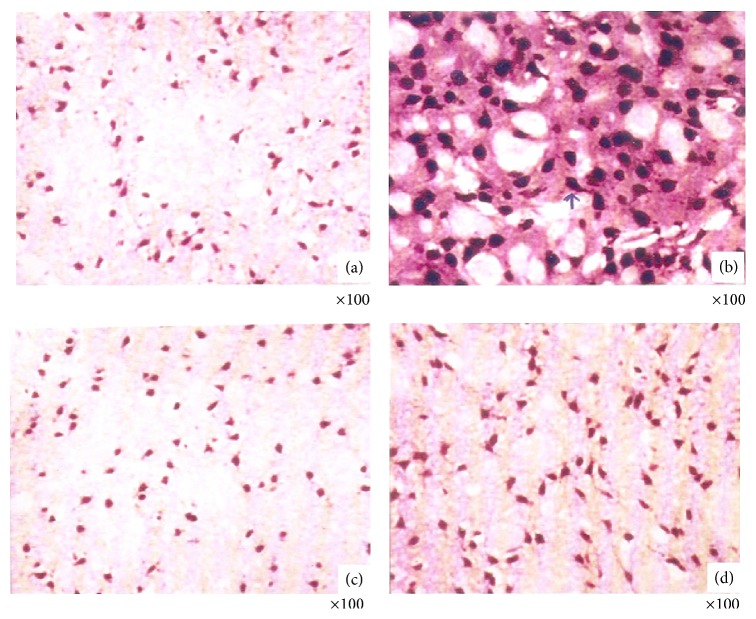
Immunohistochemical analysis of nAChR (*α*4) expression in rat brain cerebellum. (a) Control rats showing normal expression of nAChR. (b) CS rats showing increased expression of nAChR. (c) BA rats showing normal expression of nAChR. (d) CS + BA rats showing decreased expression of nAChR.

Cigarette smoking upregulates nAChR in the brain, including the common *α*4*β*2^*^ nAChR subtype [23]. In the present study, an upregulation of *α*4 subunit was evident in rats exposed to cigarette smoke (Figure 3). Chronic administration of nicotine also upregulates nAChRs [98, 99] causing an increased receptor function and sensitivity to nicotine. This results in increased trafficking of nAChRs to the cell surface, increased receptor assembly and/or maturation, or other mechanisms [100]. In smokers, abstinence from smoking normalizes the nAChR upregulation to the levels of nonsmokers [101, 102]. Similarly, commonly used treatments for smoking cessation also decrease *α*4*β*2^*^ nAChR to near normal levels as in nonsmokers. In the exploratory analyses, decreases in *α*4*β*2^*^ nAChR levels are associated with decrease in the perceived rewarding properties of nicotine [103, 104]. Hence a downregulation of *α*4 nAChR in BA treated smoke exposed rats could be associated with diminished reward from cigarettes (presumably mediated at least in part through dopamine release). Taken together, these findings indicate that the role of BA on nAChR regulation could be vital in modulating nicotine response and reward pathway in chronic cigarette smoking. However, the mechanism on how BA influences the upregulation remains to be understood.

**Figure 4:**
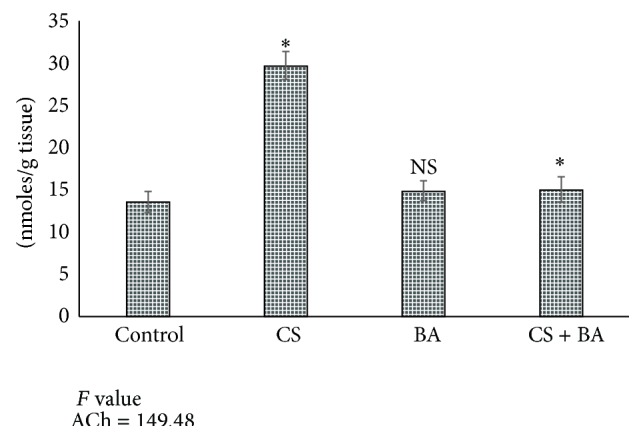
Levels of acetylcholine in brain of control and experimental animals. Values are expressed as Mean ± S.D. Significance is indicated for comparisons between control and CS and BA; Group CS versus CS + BA with Dunnett's T3 post hoc multiple comparison test; ^∗^ *P* < 0.001; NS: nonsignificant.

**Figure 5:**
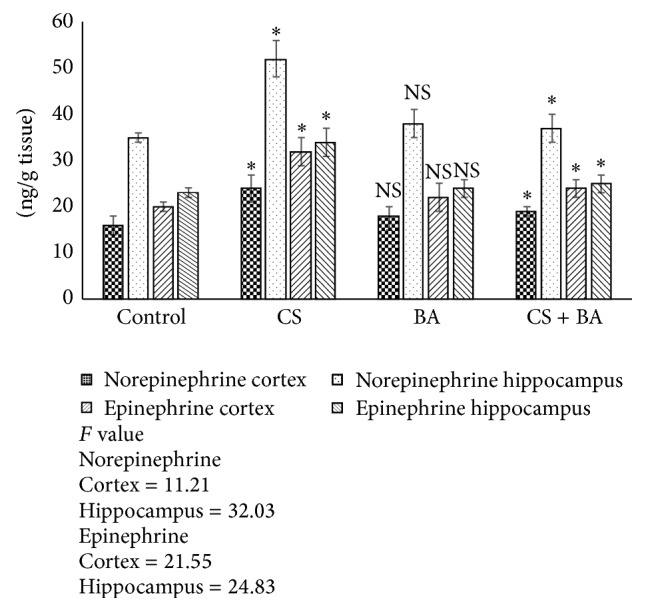
Levels of norepinephrine and epinephrine in brain of control and experimental animals. Values are expressed as Mean ± S.D. Significance is indicated for comparisons between control and CS and BA; Group CS versus CS + BA with Dunnett's T3 post hoc multiple comparison test; ^∗^ *P* < 0.001; NS: nonsignificant.

**Figure 6:**
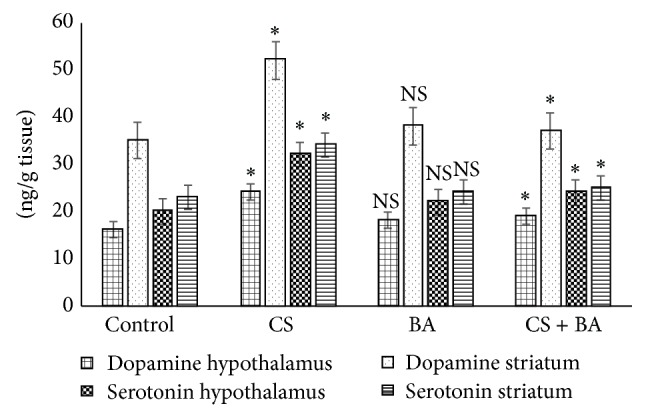
Levels of dopamine and serotonin in brain of control and experimental animals. Values are expressed as Mean ± S.D. Significance is indicated for comparisons between control and CS and BA; CS versus CS + BA with Dunnett's T3 post hoc multiple comparison test; ^∗^ *P* < 0.001; NS: nonsignificant.

**Figure 7:**
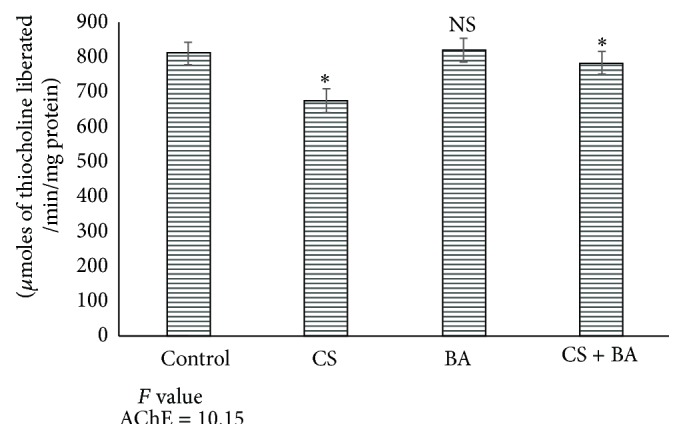
Activities of acetylcholine esterase (AChE) in brain of control and experimental animals. Significance is indicated for comparisons between control and CS and BA; CS versus CS + BA with Dunnett's T3 post hoc multiple comparison test; ^∗^ *P* < 0.001; NS: nonsignificant.

Nicotine is cholinergic by increasing the release of acetylcholine (ACh) from axonal stores and inhibits its clearance by inhibiting acetylcholine esterase (AChE) [105–107]. Increased accumulation of ACh increases the electrical activity in rat brain [107]. This accounts for the increase in most of the neurotransmitters in rats exposed to cigarette smoke (Figures 4–6). In BA treated rats, the activities of AChE were increased (Figure 7), which could have decreased the lowered levels of ACh. This confirms the anticholinergic effect of BA [108].

**Figure 8:**
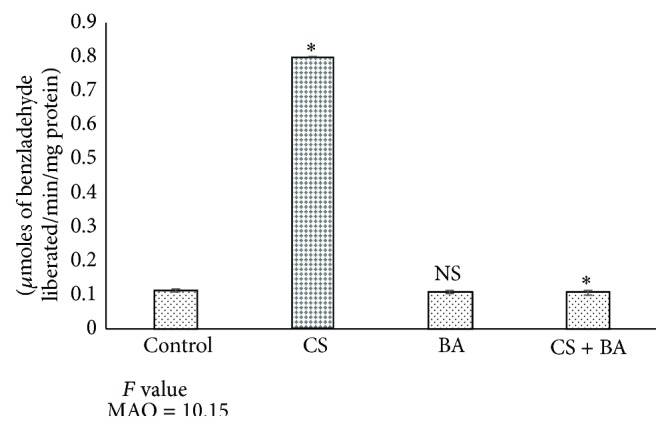
Activities of monoamine oxidase (MAO) in brain of control and experimental animals. Significance is indicated for comparisons between control and CS and BA; Group CS versus CS + BA with Dunnett's T3 post hoc multiple comparison test; ^∗^ *P* < 0.001; NS: nonsignificant.

Increases in plasma catecholamines are known to occur with smoking [109]. Upregulation of nAChR increases the release of catecholamines: epinephrine and norepinephrine, an effect mediated through the tyrosine hydroxylase activity [110]. Vasoconstrictor effects observed in smoking are related to increases in norepinephrine [111]. In the present study, smoking induced an increase in the levels of epinephrine and norepinephrine in rat brain (Figure 8). However, BA administration maintained the levels of norepinephrine in treated rats. The observed lowering could be due to the downregulation of nAChR by BA. Apart from its ability to induce downregulation of nAChR expression, BA could have interacted with tyrosine hydroxylase [112] and modulated the release of catecholamines.

Nicotine also influences the release of serotonin, and it has been reported to have a dual role as it induces both an increase and decrease [113, 114]. In the present study, cigarette smoking increased the serotonin level in rats. Serotonergic dysfunction has also been in smokers [115]. Serotonergic dysfunction is associated with clinical depression and depression is far more prevalent among smokers [116] suggesting a possible link. Further, compounds that increase dopamine and its metabolites concentration have abuse potential like opiates and cocaine, whereas those which lower dopamine induce cognitive, behavioral, and motor coordination defects [117]. The role of BA on serotonin [118] could have maintained the levels in treated animals (Figure 8). Physiologically, high level of neuronal dopamine induces greater oxidative stress derived from dopamine [119]. These results confirm the effect of* Bacopa monnieri* extract in normalizing norepinephrine, serotonin, and dopamine in cortex and hippocampus of rats, in both acute and chronic unpredictable stress [120]. In the cigarette smoke exposed rats, an increase in dopamine levels was observed, but in BA administered rats the levels were maintained at near normal. This reflects the safety and subsequent tolerability of BA in preclinical models as it did not induce any untoward and toxic effect.

Most of the nicotine-mediated release of neurotransmitters occurs via modulation by presynaptic nAChRs, although direct release of neurotransmitters also occurs [121]. Dopamine release is facilitated by nicotine-mediated augmentation of glutamate release and with long term treatment, by the inhibition of GABA release [122]. In addition to direct and indirect stimulation of neurotransmitter release, chronic cigarette smoking (but not nicotine administration) reduces brain monoamine oxidases A and B (MAO-A and MAO-B) activity, which would be expected to increase monoaminergic neurotransmitter levels such as dopamine and norepinephrine in synapses, thus augmenting the effects of nicotine and contributing to addiction [123]. Inhibition of MAO facilitates acquisition of nicotine self-administration in rats, supporting the idea that MAO inhibition interacts with nicotine to reinforce tobacco dependence [124]. Decreased activity of MAO in cigarette smoking exposed rats (Figure 8) confirms reports that have shown downregulation of MAO expression, including MAO-A and MAO-B in the brain, [125, 126] as well as influencing methylation of MAO promoter genes [127]. This lowering could have resulted in an increase in dopamine content in cigarette smoke exposed rats. Increases in MAO activities in BA treated rats (Figure 8) confirm the reports of recent studies which have shown the influences of* Bacopa monnieri* on the activities of MAO [128].

Polyamines play a key role in brain cell replication, differentiation, and regulation of nAChRs and they influence synaptic transmission [129, 130]. Alterations in polyamine gating of cholinergic synaptic signaling contribute to adverse neurobehavioral effects of numerous neuroteratogens [130]. Ornithine decarboxylase (ODC) is the rate limiting enzyme in the maintenance of polyamine levels. Inhibition of ODC inhibits growth and induces gross dysmorphology, upregulating the *α*7 and *α*4*β*2^*^ nAChR. This is accompanied by abnormalities in macromolecular indices of cell packing density and cell membrane surface area. In chronic cigarette smoking exposed rats, ODC activity increased significantly (Table 2).

Excitotoxic challenge induces neuronal proliferation and induces ODC [131]. Induction of ODC is neuroprotective in cerebral ischemia [132], and, however, is also a common response in various pathological stimuli in brain such as physical, chemical, thermal, and metabolic injuries [133]. A relatively long lasting increase in ODC and consequently its product putrescine are causally related to neurodegeneration [134]. In the present study, cigarette smoking increased the activities of ODC. BA treated rats recorded a decrease in ODC activity confirming its role in inhibiting neurodegenerative process following cigarette smoking induced excitotoxicity in brain.

### 8.3. Nicotine and Cotinine Levels

Cigarette smoking increases the levels of nicotine and its metabolite cotinine to pharmacologically active concentrations that are responsible for mediating the aspects of nicotine dependence. In rats exposed to cigarette smoke, accumulation of cotinine in brain was noted (Table 3), and the levels were lowered in BA treated rats. The decrease in the levels could have probably resulted from the increased clearance of cotinine by the CYP system. Although* Bacopa monnieri* extract reportedly inhibits CYP enzymes [135], increased clearance of cotinine, as noted from a decrease in cotinine levels in BA treated rats, confirms that purified bacosides do not inhibit CYP; instead, the constituents in crude extract exert an inhibitory effect [136, 137].

Cigarette smoking accelerates the metabolism of drugs, especially the ones primarily metabolized by CYP1A2 [138]. It delays the clearance of nicotine [139]. In smokers, nicotine clearance is increased by 14% in 4-day smoking abstinence and by 36% higher in 7-day smoking abstinence compared to overnight abstinence. Apart from nicotine, substance(s) in cigarette smoke, as yet unidentified, also affect the metabolism of nicotine. For instance, cotinine slows the metabolism of nicotine since both are metabolized by the same enzyme [140]. However, carbon monoxide in cigarette smoke has no effect on nicotine and cotinine clearance [141], but *β*-nicotyrine, a minor alkaloid in cigarette smoke, effectively inhibits CYP2A6 in vitro [142]. Thus, reduced nicotine clearance may also result from downregulation of CYP expression and not inhibition [143].

Cigarette smoking also induces glucuronidation of some drugs, such as propranolol and oxazepam, and UGT1A9 is the inducible component of 3′-hydroxycotinine O-glucuronidation [143]. Excretion of 3′-hydroxycotinine O-glucuronide is induced by smoking, but the extent of nicotine and cotinine N-glucuronidation is not significantly affected. In rats exposed to cigarette smoke increase in UDP-GT was noted and the activities remained unaltered in BA treated rats [144]. The adaptogenic role of* Bacopa monnieri* is evident from increased cotinine clearance [145].

### 8.4. Oxidative and Peroxidative Changes

Free radicals mediated oxidative stress has been implicated in the pathogenesis of smoking-related diseases and antioxidant nutrients are reported to prevent the oxidative damage induced by smoking. Cigarette smoking modulates antioxidant status in various organs by increasing lipid peroxidation and prooxidative state [146]. Increased basal and induced lipid peroxidation were observed in cigarette smoke exposed rat brain [147]. Acute exposure to cigarette smoke enhances the production of antioxidant enzymes as a result of adaptive response that mitigates the damage [148], but chronic exposure decreases the inherent antioxidant defense in brain [149, 150].

The constituents of cigarette smoke affect the individual cellular antioxidants differently. The quinone/semiquinone radicals from the tar phase of cigarette smoke inactivate superoxide dismutase [151] and inhibit catalase in brain [152]. Acetaldehyde, a major aldehyde from the smoke, depletes cell of cellular glutathione [153]. Other cellular antioxidants, tocopherols, carotenoids, and retinol, are destructed by cigarette smoke [154].

Further the cigarette tar contains large amounts of metals, complexed to some components of tar such as odiphenols [155], which can mobilize reactive iron from ferritin and copper from copper binding protein inducing damage to brain [156]. The heavy metal cadmium, in cigarette smoke, decreases the bioavailability of selenium (Se) and zinc (Zn) and thus depletes the antioxidant status [157]. The role of BA as chelator of transition metal, inhibition of free radicals, and termination of lipid peroxidation at the initiation level itself [69] accounts for its protection in cigarette smoke induced lipid peroxidative damage and combative against oxidative damage.

### 8.5. Mitochondrial Functions

Mitochondria are the site of cellular oxidation and provide ATP for various metabolic processes and hence are vulnerable to free radical attack. Mitochondrial damage is prevalent in both heart and brain following cigarette smoke exposure [158, 159]. Exposure to cigarettes can lead to mitochondrial dysfunction as demonstrated by increased levels of cholesterol, lipid peroxides, and increased cholesterol/phospholipid ratio, in conjunction with decreased mitochondrial enzymes in rats exposed to cigarette smoke [160]. Chronic cigarette smoking prevented exercise-induced improvement in brain mitochondrial function and neurotransmission [161]. Perturbed mitochondrial energetics is critical in normal brain development [6, 162]. Cerebellar perturbation can broadly impact regulation of behavioral and cognitive domains [163].

Aerobic demands increase postnatally with heighted synaptic development, requiring more ATP to maintain membrane polarity. Exposure to cigarette smoke perturbed the mitochondria and associated aerobic pathways. The effect of BA in regulating the key aerobic ATP production, probably by preventing the peroxidative changes in mitochondria, could be crucial in mitochondrial mediated neurotransmission pathways. Brain energetics is highly regulated process and further studies in the mechanistics can provide an insight into the role of BA.

### 8.6. Membrane Integrity and Electrolyte Balance

Derangement of membrane bound enzymes and modifications of lipid bilayer alterations following cigarette smoke exposure resulted in significant decrease in the activities of ATPases [164]. Free radicals in cigarette smoke deplete cell protein sulfhydryl groups and increase in protein carbonyl formation [165] and so does acetaldehyde in cigarette smoke [166]. Membrane bound ATPases are thiol-dependent enzymes, and modification of thiol groups within the active sites of these enzymes lowers their activities in cigarette smoke rats. The antioxidant role of BA prevented the membrane damage and restored the activities of ATPases. Also the restitution of ATP levels by altering the mitochondrial dysfunction maintained the activities of ATPases.

Inhibition of Na^+^/K^+^-ATPase and elevation of Na^+^ in chronic exposure to cigarette smoke are attributed to the increased cholesterol/phospholipid ratio [167] followed by neuronal apoptotic death mediated by intracellular depletion of K^+^ and accumulation of Na^+^ and Ca^2+^ [168]. Plasma membrane Ca^2+^-ATPase (PMCA) is a regulator of intracellular calcium which undergoes early developmental changes in rat brain as a function of its maturity [169]. PMCA is very sensitive to the inhibitory effect of reactive oxygen species (ROS) due to the age dependent oxidative modification of PMCA and the related chronic oxidative stress [170].

In addition to generation of free radicals, cellular degeneration that is involved in cigarette smoking is related to the accumulation of advanced glycosylation end-products (AGE). Activities of several enzymes are inhibited due to enzyme protein glycation [171, 172]. The changes in the Ca^2+^ ATPase can be related to the increased glycation found in cigarette smoke exposed rats that in turn may lead to the enzyme protein glycation [173]. Alterations in the capacity to maintain normal calcium homeostasis underlies the reduced cellular function bound with the aging process. In the brain, multiple methionines within the calmodulin molecule become oxidized to methionine sulfoxides, resulting in an inability to activate a range of target proteins, including plasma membrane Ca^2+^-ATPase [174].

Mg^2+^-ATPase is not uniformly distributed and differs in respect to affinity for ATP in rat brain regions [175] and is activated by millimolar concentrations of Mg^2+^. Comparison of Na^+^, K^+^-ATPase, and Mg^2+^-ATPase activities in the synaptic plasma membrane from various regions of rat brain reveals that moderate hypoxia increases the activity of synaptosomal Mg^2+^-ATPase whereas activities of both Ca^2+^- ATPase and Na^+^, K^+^-ATPase are decreased [176].

Increased concentrations of Ca^2+^ by stimulating Na^+^/Ca^2+^ exchanger produce cellular Mg^2+^ depletion since excessive calcium displaces magnesium from its binding sites [177]. Decrease in Mg^2+^ in turn inhibits Na^+^/K^+^-ATPase further, as ATP-Mg complex is the actual substrate for the enzyme [178]. Rats exposed to cigarette smoke showed a decrease in the activity of brain Mg^2+^-ATPase. The restoration of membrane bound ATPases maintained the electrolyte homeostasis in brain, impairing electrolyte balance in cigarette smoking.

### 8.7. Apoptotic and Neurogenic Changes

Dysregulation of apoptosis is an important factor in the pathogenesis of cigarette smoking [179]. Nicotine is involved in both stimulation and inhibition of neuronal apoptosis [180–182]. Apoptosis is suggested as a possible contributing factor in the pathogenesis of smoking-induced toxicity. Exposure to cigarette smoke induced apoptosis as characterized by DNA laddering, increased TUNEL-positive cells, and apoptotic features evident ultrasctructurally in the brain. Administration of BA prevented expression of hsp70 and neuronal apoptosis during cigarette smoking [183]. Extract of BM reduced oxidative stress by improving Nrf2 expression and results in improvement in antiapoptotic (Bcl2) expression and decreased proapoptotic (Bax and caspase-3 activity) indicating neuroprotection [184].

### 8.8. Therapeutic Implications of BA in Passive Smoking

An insight into these observations supports the role of BA as a supplement for secondhand smoking. Its role on nAChR expression may underpin its effect on cigarettes induced neurochemical alteration. Generally antidepressants are noncompetitive inhibitors of nAChRs [185] and so it is possible that the role of BA as a noncompetitive inhibitor to nAChRs could potentially help in controlling the nAChR mediated upregulation of neurotransmitters and nicotine dependence [186], apart from its role on nAChR expression at the transcriptional level.

Other potential sites of action for BA worthy for consideration include its ability to control inflammation and oxidative stress. Antioxidants and anti-inflammatory drugs potentially negate the anxiolytic behaviors [187, 188], a feature also prevalent in passive smokers. Exploitation of the antioxidant property of BA could aid in overcoming oxidative anxiety disorders.

## 9. Conclusion

A number of admonitions exist in the data presented. The interpretations are drawn from a study involving chronic exposure of rats to cigarette smoke and not acute cigarette smoke. The cross-sectional nature of this work is hampered from conclusions not drawn from molecular pathways. Future research efforts in this area should attempt to address these shortcomings. It would be useful to ascertain the effects of BA on individual components of cigarette smoke constituents involving multiple pathways. Given that passive smoking affects multiple pathways and may increase risk of developing anxiety, triangulation of potential effects involving a combination of animal and human models will likely be required. As the role of BA appears to be multifaceted, it may represent a future therapeutic means for secondary smoke. In addition, to its neuroactive role, BA as an anti-inflammatory and antioxidant agent may assist in improving the symptoms, as they may do in other conditions pertaining to oxidative stress. Further studies addressing this area may elicit insights into new therapeutic opportunities.

## Figures and Tables

**Table 1:** Constituents of the cigarette smoke.

Smoke constituents	Concentration/cigarette
Nicotine	1.8 mg
Carbon monoxide	20 mg
Total particulate matter	32 mg
Acetaldehyde	0.9 mg
Hydrogen cyanide	225 mg
Benzene	38 mg
N'nitrosonorcotine	240 mg

**Table 2:** Levels of nicotine and cotinine in brain of control and experimental animals. Values are expressed as Mean ± S.D.

Parameter	Control	Cigarette smoke (CS)	Bacoside A (BA)	Cigarette smoke + bacoside A (CS + BA)	*F* value
Nicotine (ng/g tissue)	n.d	180 ± 12	n.d	89 ± 5^*^	870.08
Cotinine (ng/g tissue)	n.d	210 ± 15	n.d	120 ± 8^*^	718.14

Significance is indicated for comparisons between control and CS and BA; CS versus CS + BA with Dunnett's T3 post hoc multiple comparison test; ^∗^ *P* < 0.001.

n.d: not detected.

**Table 3:** Activities of ornithine decarboxylase in brain of control and experimental animals. Values are expressed as Mean ± S.D.

Parameter	Control	Cigarette smoke (CS)	Bacoside A (BA)	Cigarette smoke + bacoside A (CS + BA)	*F* value
Ornithine decarboxylase nM of ^14^CO_2_ released/hr/g tissue	2.0 ± 0.12	5.65 ± 0.52	2.23 ± 0.22	2.45 ± 0.023^*^	3489

Significance is indicated for comparisons between control and CS and BA; Group CS versus CS + BA with Dunnett's T3 post hoc multiple comparison test; ^∗^ *P* < 0.001.

n.d: not detected.
